# Assessment of Constipation in Patients With Cancer

**Published:** 2016-05-01

**Authors:** Rita J. Wickham

**Affiliations:** Rush University College of Nursing (Adjunct Faculty), Chicago, Illinois

Constipation, a significant problem for many cancer patients, often leads to significant physical and psychological distress. Clinicians must recognize constipation in a timely manner to optimize management and minimize its adverse effects (Andrews & Morgan, 2013). There is no single accepted definition of constipation, which patients and clinicians often view differently (Clark, Urban, & Currow, 2010). For example, in one small study of cancer patients undergoing palliative care, patient ratings of constipation severity were influenced more by ease of passing stool than frequency of bowel movements or stool consistency (Brown, Lawrie, D’Sa, Wilcox, & Bennett, 2006; McCrea et al., 2008). Patients may say they are constipated if they experience any of several changes in bowel movements, whereas clinicians usually view constipation as hard and infrequent stools (Izumi, 2014).

Other authors have reviewed constipation assessment tools and generally agree that constipation is subjective and should be assessed by patient report. Instruments should capture the severity of constipation as well as the quality-of-life effects from it. Although there are no agreed-upon instruments to assess constipation in cancer patients, clinically useful tools would balance the length and complexity with information provided to minimize patient burden and gather useful data to evaluate constipation severity and direct management. Although others are available, the patient-rated scales selected for this review are valid, brief, and clinically useful.

## THE CONSTIPATION ASSESSMENT SCALE

**Figure 1:**
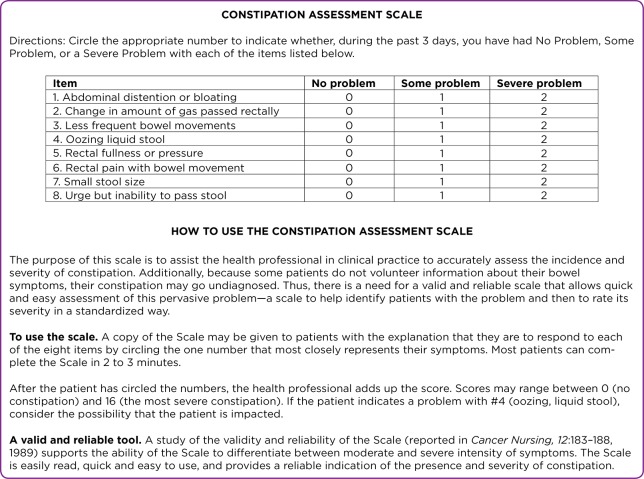
Constipation Assessment Scale. Used with permission from Susan C. McMillan, PhD, RN, Professor, University of South Florida College of Nursing, Tampa, Florida.

McMillan and Williams (1989) developed the Constipation Assessment Scale (CAS) to evaluate the constipation cancer patients experienced during the past week. The CAS (Figure 1) was based on earlier research and clinical literature and includes eight commonly identified characteristics of constipation, including fewer bowel movements, smaller bowel movements than deemed "normal," and difficult or painful bowel movements (Figure 1). The CAS discriminated between patients constipated secondary to receiving vinca alkaloids or opioids and healthy adults who were not constipated, supporting its validity. Similarly, the CAS discriminated between moderate and severe constipation in patients given a vinca alkaloid 3 weeks earlier and those taking morphine, supporting its construct validity.

The CAS has good internal consistency (r = 0.7–0.78) and high test-retest coefficients (r = 0.98), providing strong evidence for its reliability. It takes patients about 2 minutes to complete and is formatted at a 6th-grade reading level. The patient rates each constipation item on a three-point scale (no problem to severe problem). Total scores range from 0 (no constipation) to 16 (worst possible constipation). No cutoff score for constipation has been reported.

The CAS was used in another small study (n = 46) to evaluate its psychometric properties in assessing constipation in a convenience sample of pregnant women compared with healthy women of childbearing age and nursing students (Broussard, 1998). The CAS was found to have acceptable content validity (expert panel review agreement 0.75, Cohen’s kappa 0.714) to assess constipation in this population. Reliability was evaluated by test-retest in 16 nursing students, with high correlations (r = 0.84–0.92), and internal consistency for pregnancy in the 30 pregnant women was á = 0.82.

## THE PATIENT ASSESSMENT OF CONSTIPATION

**Figure 2:**
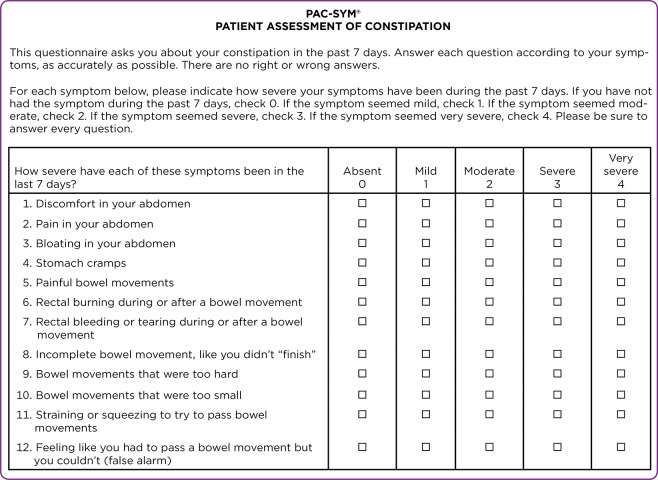
Patient Assessment of Constipation—Symptoms. Adapted with permission from Mapi Research Trust © 1999.

The Patient Assessment of Constipation (PAC), which is shown in Figure 2, was initially developed to measure adult patients’ perspectives of chronic idiopathic constipation over time (Frank, Kleinman, Farup, Taylor, & Miner, 1999). The PAC is a self-report instrument with two complementary parts: the Symptom Questionnaire (PAC-SYM) and the Quality-of-Life Questionnaire (PAC-QOL). The PAC-SYM and the PAC-QOL can be used alone or together. The PAC-SYM is most widely used and will be discussed. The PAC-QOL also has sound psychometric properties; it captures information about patient-assessed burdens of constipation, including associated physical and psychosocial discomfort, worries and concerns, and satisfaction (Dubois, Johnson, de la Loge, & Marquis, 1998; Marquis, de La Loge, Dubois, McDermott, & Chassany, 2005).

The original (44-item) PAC-SYM was developed based on the literature and information generated from patient focus groups (Frank et al., 1999). It assessed constipation frequency and severity (divided into two levels) by patient self-report. The final version has 12 items that fall into 3 subscales: stool, rectal, and abdominal symptoms, as well as a question about bowel movement frequency in the past 7 days. Patients rate symptoms on a 5-point (0–4) Likert scale, and the total score can range from 0 to 48.

The final PAC-SYM was validated in adults with chronic idiopathic constipation and demonstrated high internal consistency (Cronbach á = 0.89) and test-retest reliability. After treatment of constipation, responders had significantly lower PAC-SYM scores than nonresponders, indicating the PAC-SYM can distinguish groups based on symptom severity. It takes 4 to 6 minutes to complete the PAC-SYM. The authors concluded that the PAC-SYM is a highly reliable, valid, and comprehensive means to assess the effectiveness of constipation therapy in adults.

The PAC-SYM was also validated in 677 patients with non–cancer-related pain started on escalating doses of oral morphine or transdermal fentanyl and thus at risk for opioid-induced constipation (Slappendel, Simpson, Dubois, & Keininger, 2006). The PAC-SYM was sensitive, valid, and reliable at identifying opioid-related constipation; constipated patients had significantly higher mean PAC-SYM scores than nonconstipated patients.

## THE VICTORIA BOWEL PERFORMANCE SCALE

**Figure 3:**
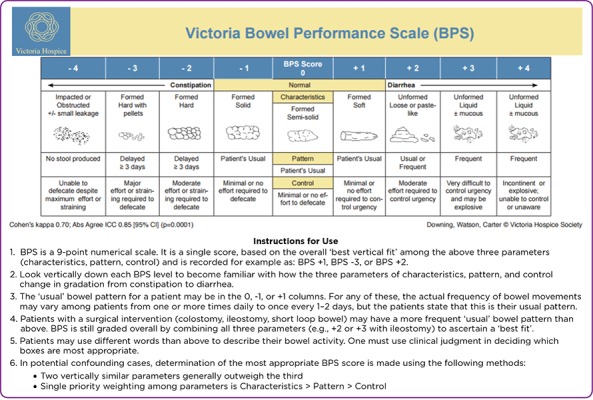
Victoria Bowel Performance Scale. Used with permission from the Victoria Hospice Society.

The Victoria Bowel Performance Scale (BPS) is a patient-centered assessment tool of bowel function and is the only tool that evaluates constipation and diarrhea (Downing, Kuziemsky, Lesperance, Lau, & Syme, 2007). The BPS (Figure 3) is intended to rapidly and easily assess changes in bowel status, particularly in palliative care patients. This bipolar, 9-point ordinal scale ranges from –4 (constipation) to +4 (diarrhea) and includes 3 assessment parameters: stool frequency, consistency, and the patient’s ability to control evacuation (Downing et al., 2007). The health professional collaborates with the patient to complete the BPS to reach a single score based on the overall ’’best vertical ﬁt’’ in the assessment parameters.

The BPS was ﬁeld tested among nurses and physicians to assess content validity, utility, accuracy, and ease of use (Downing et al., 2007). Inter- and intrarater reliability was evaluated by a test-retest design in a convenience sample of palliative care staff nurses and clinical nurse specialists, home care nurses, oncology nurses, and physicians who used the BPS to rate constipation in web-based test-case scenarios (12 of 18 cases involved cancer patients) at 1-week intervals. Intraclass correlation (n = 54) for both time periods was 0.828 (confidence interval, 0.728–0.916).

Hawley and colleagues (2011) assessed the usefulness of the BPS to audit outpatient oncology symptom management clinics, palliative care units, and residential hospices before and after orientation to and implementation of a constipation-monitoring program. Clinicians considered the BPS acceptable and easy to use, and after implementation, documentation and laxative prescriptions increased from 33% to 69% of visits and 16% to 39%, respectively (*p* < .001; Hawley, Barwich, & Kirk, 2011).

## SINGLE-ITEM PATIENT-RATED CONSTIPATION MEASURES

Although the CAS, PAC-SYM, and BPS all assess more than one dimension of constipation, it might be useful to have a single-item tool that could be incorporated into a symptom screening tool for all patients, particularly because constipation is underassessed and leads to significant psychological and physical consequences in so many patients. The primary aims of a study by Rhondali and colleagues (2013) were to compare the accuracy of a patient-reported constipation (PRC) scale with modiﬁed Rome III criteria (the most widely used criteria to define functional constipation) and agreement between the PRC and a yes-or-no question about being constipated.

The PRC format is familiar to most clinicians: The patient is asked to rate a symptom on an 11-point numeric rating scale, which can be used in a verbal or written format (Rhondali et al., 2013). The clinician asks a patient to rate a symptom from 0 (no symptom—not constipated) to 10 (worst possible symptom—worst possible constipation). The investigators concluded that asking patients to rate constipation on the numerical scale is sensitive and specific, and a rating of ≥ 3 identifies constipated patients. Conversely, merely asking patients if they are constipated (yes/no) is not clinically useful, because it misses almost one-third of patients with constipation.

Similarly, another study included patients with advanced cancer who were using constipation interventions (laxatives, suppositories, enemas, Chinese herbal medicine, or digital evacuation) or rated their constipation as ≥ 2 on a 0 (none) to 7 (most severe) verbal descriptor scale (Cheng, Kwok, Bian, & Tse, 2013). The majority of the 225 patients in this study were constipated, with 50.7% reporting mild (2–4) and 29.8% reporting severe (5–7) constipation. Even some patients having daily bowel movements reported constipation. The authors concluded that patients’ perceptions of constipation are influenced by their experiences and what they deem "normal" bowel movements, which may not be congruent with a clinician’s assessment.

## SUMMARY

Valid and reliable measures of constipation are useful to screen for constipation, as well as to form the basis for a more thorough constipation assessment, interventions, and evaluation. The CAS and the PAC-SYM are minimally burdensome to patients and useful to practitioners. The Victoria BPS may be more useful for patients with advanced cancer but has a normative range for bowel movements, which may be clinically useful in other settings. Single verbal descriptor scales (0–7 or 0–10) may identify the severity of constipation (by patient perception) and also may guide the aggressiveness of interventions for constipation. Each of these tools could be useful to advanced practitioners in different clinical settings.
